# Evaluating equity, diversity, and inclusion in Canadian Postgraduate Medical Education: A cross-sectional analysis of online content

**DOI:** 10.1371/journal.pone.0307584

**Published:** 2024-08-27

**Authors:** Mohamed Bondok, Mostafa Bondok, Liana Martel, Christine Law

**Affiliations:** 1 Cumming School of Medicine, University of Calgary, Calgary, AB, Canada; 2 UBC Faculty of Medicine, University of British Columbia, Vancouver, BC, Canada; 3 Faculty of Medicine, University of Ottawa, Ottawa, ON, Canada; 4 Department of Ophthalmology, School of Medicine, Queen’s University, Kingston, Ontario, Canada; BOKU: Universitat fur Bodenkultur Wien, AUSTRIA

## Abstract

**Background:**

Medical graduates applying to Residency through the Canadian Resident Matching System (CaRMS) utilize the internet to gather information on programs and their overarching Postgraduate Medical Education (PGME) Office. This study aims to evaluate how PGME websites across Canada convey their commitment to equity, diversity, and inclusion (EDI) through their website features.

**Methods:**

Cross-sectional analysis of the 17 Canadian PGME websites against 20 EDI criteria based on contemporary literature, across five domains: leadership and governance, recruitment, accommodations, community engagement, and pathways to entry. Non-parametric testing was conducted to explore the relationship between EDI performance and municipal population diversity and geographic region.

**Results:**

The evaluation of PGME websites, policies, reports, and plans revealed a mean score of 8.65/20 (SD = 3.00), with scores ranging from a minimum of 4/20 to a maximum of 13/20, indicating variability in EDI representation. Specifically, the domain of leadership and governance demonstrated the highest mean proportion of completed criteria (51%), while community engagement had the lowest (24%). Notably, 9 out of 17 PGME websites (53%) met at least 10 EDI criteria. Analysis by geographic region demonstrates significant mean differences (p = 0.02), with Ontario (10.50, SD = 2.17) and Western Provinces (11.00, SD = 0.00) scoring notably higher than Quebec (4.50, SD = 0.58), the Prairies (8.50, SD = 2.12), and the Atlantic region (8.00, SD = 2.83).

**Conclusions:**

The assessment of Canadian PGME websites reveals varying levels of commitment to EDI. While many programs exhibit strong EDI representation in mission statements, access to mental health services, and anti-discrimination policies, there are notable gaps in leadership messaging, diverse interview panels, family-friendly policies, and deliberate recruitment of underrepresented groups. Regional differences highlight the need for sharing best practices to promote inclusivity across the country. Improving EDI efforts on PGME websites can promote the recruitment and retention of a diverse resident population.

## Introduction

Equity, diversity and inclusion (EDI) plays a vital role in healthcare by promoting equitable access to care and improving patient outcomes [1, 2]. Fostering a diverse healthcare workforce effectively reduces barriers to care, such as language and cultural differences. Diversity also creates learning environments that promote empathy, critical thinking, and broader perspectives among healthcare professionals [3, 4].

In recent years, medical schools across Canada have acknowledged the importance of EDI and have taken significant steps to address inequities in medical school admissions. Initiatives such as an accelerated pathways to entry, contextualized evaluations of non-academic pursuits, and sociodemographic representation of community interview panelists have contributed to this progress [5–7]. Despite the focus on EDI initiatives in undergraduate medical education, the extent to which these principles are upheld in postgraduate medical education (PGME) programs remains uncertain.

The Best Practices in Applications & Selection Working Group (BPAS) report, led by the University of Toronto PGME office, responded to provincial and national calls for increased diversity among the physician workforce to meet population health needs. This report identified gaps in the selection and retention processes, particularly for economically disadvantaged, Indigenous, and rural applicants. Recommendations included promoting diversity in the selection criteria and process, pursuing, and measuring diversity across PGME programs, ensuring selection teams have diverse perspectives aligned with program goals, and providing comprehensive training for assessors [8]. These shortcomings were acknowledged, and in 2018 the recommendations received endorsement from PGME institutions nationwide [9]. Research has demonstrated that individuals from marginalized communities, including women, those identifying as LGBTQI2S+, and other underrepresented minorities prioritize program diversity during program selections [10, 11]. Thus, the exhibition of a strong dedication to EDI by PGME to attract a diverse cohort of resident physicians is essential.

Reviews have highlighted the increasing reliance of prospective resident physicians on websites as a primary source of information when evaluating training programs and making career decisions [12, 13]. This reliance may be further amplified by restrictions on the number of visiting electives Canadian medical students can complete, and a shift towards virtual interviews for the Canadian residency match process [14, 15]. With students needing to make judgments on programs and institutions they have little to no direct interaction with, websites become indispensable tools for gathering essential insights and forming initial impressions. While numerous studies have examined EDI initiatives within specific medical specialties including ophthalmology, orthopedic surgery, and general surgery, there lacks a comprehensive assessment of EDI within the overarching PGME governance [16–20]. This study evaluates how Canadian PGME programs convey their commitment to EDI through website features, policies, and leadership reports.

## Methods

A list of all Canadian Faculties of Medicine was compiled using the Royal College of Physicians and Surgeons of Canada website [21]. Each institution’s PGME website was identified and subsequently reviewed. All website data was collected in February 2023 by two independent reviewers. This study was exempt from requiring ethics by the Queen’s University Health Sciences and Affiliated Teaching Hospitals Research Ethics Board (HSREB) as it relied exclusively on publicly available information.

### EDI evaluation criteria

All Canadian PGME websites were evaluated based on EDI criteria adapted from the Diversity Audit Tool (DAT) [22]. Initially created by the Ryerson University Diversity Institute and the Canadian Advanced Alliance Women in Technology to boost women’s participation in the information and communications technology sector, the DAT was later refined to include education and healthcare sectors [22]. The DAT examines how organizations leverage EDI across their value chain through 58 dichotomous assessment criteria. Compared to six other tools, including the Canadian Bar association equity and diversity guide, and the chamber of minerals and energy Australia gender diversity audit tool, the DAT distinguishes itself for its comprehensiveness and macro-level approach, enhancing its adaptability across various industries and sectors [22].

When relevant, assessment criteria language was adjusted to align with the study context. For instance, the inquiry "Has the business case for diversity been developed and widely communicated?" was refined to "Are the benefits of diversity communicated anywhere on the PGME website?" Similarly, elements of the DAT that could not be fairly assessed on webpages, such as "Does succession planning take into account diversity targets?" were excluded. The reasons for exclusion, detailed in **S1 Table**, include assessment challenges, limited information disclosure, and lack of applicability. These exclusions were considered in the broader context of literature reviews on program discipline EDI reviews, ensuring relevance to medical education and the Canadian landscape [16–20, 23]. The 20 retained EDI criteria were subsequently grouped into five distinct themes, as listed in **Table 1**.

**Table 1:** Description of EDI criteria assessed on PGME webpages.

EDI Criteria	Definition of Criteria
**Theme One: Leadership and Governance**	
a) Mission	Does the PGME consider diversity in identifying and developing trainees?
b) Associate Dean & Chair	Do senior leadership members (i.e., associate dean, chair) proactively communicate the importance of diversity through written statements?
c) Diversity Council	Is there a diversity council or related diversity-promoting organization or voice?
d) Director or VP Diversity	Is there a Diversity Officer at the Senior VP or Director level with lines of authority?
e) Diversity Promotion	Are the benefits of diversity communicated anywhere on the PGME website?
f) Explicit Diversity Goals	Are explicit diversity goals and policies in place and communicated?
g) Harassment / Discrimination	Are there well-developed mechanisms (i.e., policies, procedures) to handle trainee complaints about harassment and discrimination?
**Theme Two: Transparent Recruitment Practices**	
a) Underrepresented Groups	Do selection committees specifically target underrepresented groups?
b) Representative Committee	Are the selection committee’s representative (3+ different roles/positions or 1+ non-physician)?
c) Bias-free Interviewing	Are bias-free interviewing processes promoted and used?
d) Diversity Reporting	Is accountability for diversity targets and practices built into evaluating the performance of the PGME?
**Theme Three: Program Accommodations**	
a) Flex work Policy	Are flexible working arrangements available?
b) Family-friendly Policy	Are family-friendly policies in place (i.e., extended parental leave, family emergency days, elder care, and support for parents traveling)?
c) Emergency Daycare Onsite	Are on-site childcare and emergency day care services available?
d) Workload Management	Are trainee workload and program expectations managed? Are fatigue and level of supervision considered?
e) Coaching/Counselling	Do trainees have access to coaching and counselling to help manage workload and stress?
**Theme Four: Community Engagement**	
a) Newsletters	Is the importance of diversity communicated in any publicly available publications from the last 12 months?
b) Community initiatives	Is the importance of diversity and health equity considered and communicated through publicly shared community engagement activities or partnerships?
**Theme Five: Pathways to Entry**	
a) Diversity Advocacy	Does the PGME participate with external or internal associations and professional organizations in programs to promote its commitment to diversity?
b) Re-entry and Transition Programs	Does the PGME collaborate and encourage development of re-entry and transitional programs?

EDI: equity, diversity, and inclusion. PGME: postgraduate medical education.

Each criteria was assigned one point. For inclusion, information had to be available directly on the PGME website, through hyperlinks on the landing page, or in publicly accessible linked documents (**S2 Table**). Two investigators (MSB and MB) independently evaluated the presence or absence of EDI criteria, and discrepancies were resolved by consensus. French websites were evaluated by a bilingual reviewer (LM), then electronically translated to English for evaluation by a second reviewer (MSB).

### Subgroup analysis

Subgroup analysis comparing EDI scores based on geographic location and municipal population diversity was conducted. For geographical location, programs were grouped into six regions based on Statistics Canada standardized segmentation: Atlantic (Nova Scotia, Newfoundland and Labrador); Quebec; Ontario; Prairies (Saskatchewan and Manitoba); and Western Provinces (Alberta and British Columbia) [24]. Municipal population diversity was assessed using the 2022 Canadian Census data on ethnocultural diversity of each program’s respective metropolitan area, based on their listed corresponding address [25].

### Statistical analysis

Descriptive statistics were used to report website EDI scores. Kruskal-Wallis tests were conducted to assess for differences in mean EDI scores based on municipal population diversity and geographic location segmentation. Post hoc tests for pairwise comparisons were performed using Dunn’s post-hoc test with Bonferroni correction. Statistical analyses were performed using SPSS Statistics version 28 (IBM Corporation). P values less than 0.05 were considered statistically significant.

## Results

**Fig 1:**
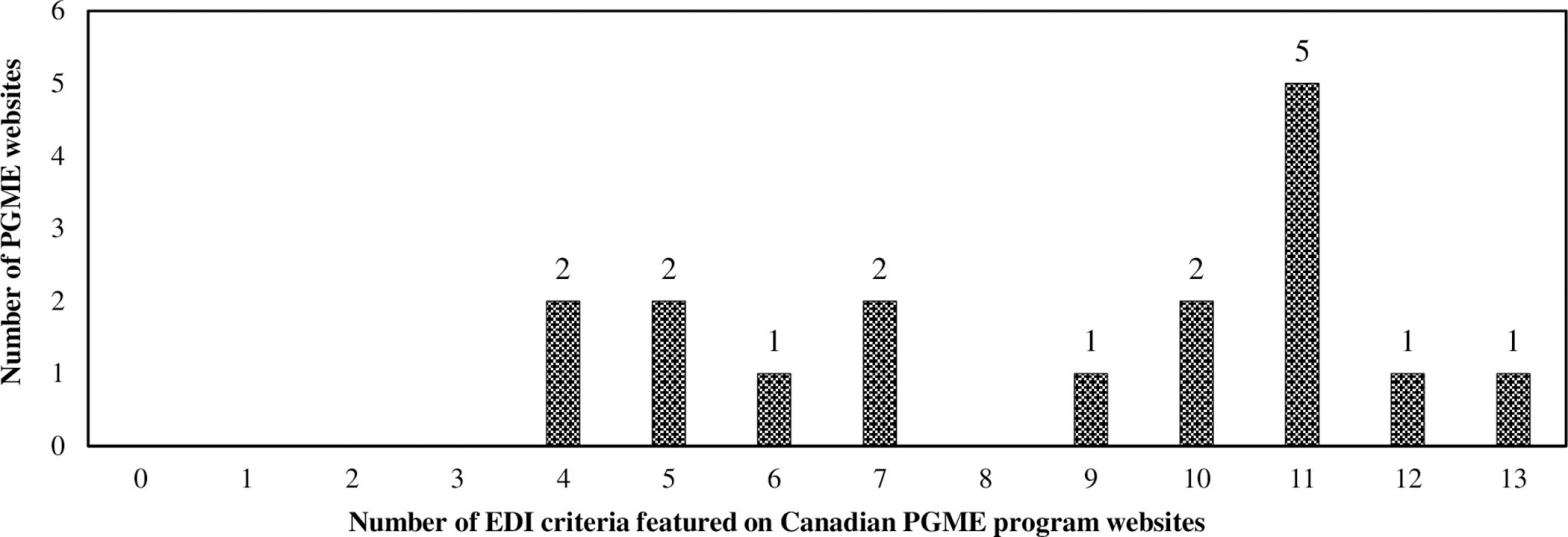
Distribution of total EDI criteria present on Canadian PGME websites. A total of 20 criteria were assessed across 17 PGME webpages, showing the number of programs meeting the specific number of EDI criteria of the total evaluated. PGME: Postgraduate Medical Education, EDI: equity, diversity, and inclusion.

A total of 17 PGME websites were evaluated, along with their associated policies, annual reports, and strategic plans. The mean score achieved by PGME websites was 8.65/20 (SD = 3.00). The scores ranged from a minimum of 4/20 (20%) to a maximum of 13/20 (65%) (Fig 1). Among the evaluated webpages, 9/17 (53%) successfully met at least 10 EDI criteria (Fig 1). When categorizing the criteria by grouped themes, the leadership and governance theme had the highest mean proportion of completed EDI criteria, with 3.6/7 (51%) criteria, while diversity in community engagement was lowest, with 0.5/2 (24%) criteria.

A total of 14 (82%) PGME websites mentioned EDI in their mission statement, while less than a third (29%) of deans or equivalent institutional leadership mentioned EDI in their individualized messages **(Table 2)**. A total of 1/17 (6%) PGME websites discussed emergency daycare onsite, and only 3/17 (12%) mentioned family-friendly policies that support trainees balancing other familial commitments **(Table 2)**. Conversely, harassment/discrimination policies and access to coaching/counselling were mentioned on all PGME websites (100%), as demonstrated in **Table 2**.

**Table 2:** EDI criteria on Canadian Post-graduate Medical Education websites by theme.

EDI Criteria	No. of Programs (%)
**Theme One: Leadership and Governance**	
a) Mission	14 (82)
b) Associate Dean & Chair	5 (29)
c) Diversity council	9 (53)
d) VP diversity or senior	4 (24)
e) Diversity promotion	6 (35)
f) Explicit diversity goals	7 (41)
g) Harassment/Discrimination	16* (94)
**Theme Two: Transparent Recruitment Practices**	
a) Underrepresented groups	5 (29)
b) Representative committee	3 (18)
c) Bias-free interviewing	11 (65)
d) Diversity reporting	4 (24)
**Theme Three: Program Accommodations**	
a) Flex work policy	9 (53)
b) Family-friendly policy	2 (12)
c) Emergency daycare onsite	1 (6)
d) Workload management	10 (59)
e) Coaching/counselling	17 (100)
**Theme Four: Community Engagement**	
a) Newsletters	5 (29)
b) Diversity programs/donations	3 (18)
**Theme Five: Pathways to Entry**	
a) Diversity advocacy	2 (12)
b) Re-entry and transition programs	14 (82)

EDI: Equity, diversity, and inclusion. PGME: Post-graduate medical education.

*Point lost since policy hyperlink present would lead to a “404: Page not found” error upon selection. A total of 20 criteria were assessed across 17 PGME programs.

PGME scores were stratified by geographic location and municipal population diversity to examine differences in meeting the established EDI criteria (**Table 3**). Differences in scores based on geographic location were significant (p = 0.020, Kruskal-Wallis), with post hoc tests for pairwise comparisons showing significantly higher mean scores for Ontario (10.50, SD = 2.17) compared to Quebec (4.50, SD = 0.58) (p = 0.036). There were no statistically significant differences found based on regional visible minority proportion (p = 0.142, Kruskal-Wallis).

**Table 3:** EDI criteria on Canadian Post-graduate Medical Education websites by geography and population diversity.

Program Characteristics	Mean EDI Criteria (SD)	p value
**Geographic Region**		
Atlantic	8.00 (2.83)	0.020
Quebec	4.50 (0.58)	
Ontario	10.50 (2.17)	
Prairies	8.50 (2.12)	
Western	11.00 (0.00)	
**Municipal Population Diversity**		
< 10% Minority	6.50 (3.11)	0.142
10–20% Minority	9.50 (0.71)	
20–30% Minority	7.67 (3.20)	
> 30% Minority	11.20 (1.10)	

EDI: Equity, diversity, and inclusion. SD: Standard deviation. Regional visible minority proportion based on the 2022 Canadian Census data on self-reported visible minority status of each program’s respective city. Atlantic = Nova Scotia, Newfoundland and Labrador. Prairies = Saskatchewan, Manitoba. Western = Alberta, British Columbia.

## Discussion

This review of Canadian PGME websites’ commitment to EDI sheds light on the nuanced landscape of PGME websites and their alignment with the pursuit of a more diverse and inclusive medical workforce.

Our findings indicate that while the majority of PGME websites incorporate elements of EDI, there remains a gap between acknowledgment and implementation. For example, while many programs prominently feature EDI in their mission statements and have robust policies addressing harassment and discrimination, fewer explicitly outline diversity goals and policies or report on diversity target performance. This presents an opportunity for PGME programs to further embed EDI principles into their organizational structures and strategic planning processes.

Moreover, we observed regional differences in EDI website content, with programs in Western Canada showing higher scores compared to other regions. This underscores the importance of sharing best practices across geographical boundaries to foster greater inclusivity nationwide. Additionally, given no score differences were found based on regional visible minorities, institutions should analyze local community characteristics, incorporate insights into their websites, and prioritize updates to provide the latest policy information. Delving into specific domains, we find both strengths and areas for improvement.

### Leadership & governance

While PGME programs have integrated EDI into their mission statements and developed robust harassment/discrimination policies, there is room for improvement, particularly in securing written commitments to EDI from senior leadership and establishing dedicated roles focused on EDI. Additionally, less than half of the programs communicate explicit diversity goals and policies. Commitment to EDI, along with actionable steps, are essential for driving organizational change, and measuring performance is critical for assessing the effectiveness of interventions [26, 27]. Therefore, we suggest that programs include explicit EDI goals in their strategic planning reports and share both qualitative and quantitative measures of progress toward these goals.

### Transparent recruitment practices

Despite most PGME websites advocating for bias-free interviewing, there remains gaps in targeting underrepresented groups in recruitment efforts and a limited call for diverse representation on interviewing committees. Research demonstrates biases throughout application processes, which include linguistic differences in personal statements based on gender and ethnic groups, to interviewers favoring candidates who resemble them in appearance and experiences [28–30]. Additionally, the transition to virtual residency interviews may exacerbate bias, disproportionately impacting underrepresented groups [31].

Some individual programs have implemented equity-driven recruitment and selection processes, such as those for Indigenous applicants in various provinces or for Black applicants to internal medicine at the University of Toronto. However, these efforts remain limited and are often restricted to specific specialties [32, 33], and are fewer in number than those in place for medical school admissions [5–7]. PGME programs should encourage all specialties to adopt key strategies for recruitment, emphasizing explicit targeting of underrepresented groups and integrating diverse representation on recruitment panels in accordance with Governmental best practices [34]. Programs may benefit from consulting the University of Calgary’s "PGME Anti-Racism Task Force Guidance for Selection Interviews" for an example of implementation of these principles [35].

### Program accommodations

All programs mentioned access to counselling and mental health services, a crucial support given the high rates of burnout among medical trainees [36]. According to a 2021 survey by the Canadian Medical Association, burnout rates among medical trainees reached as high as 53% [37]. While most programs had a flexible work policy, the specific circumstances for granting part-time equivalent positions were often unclear. To provide context, an estimated 15% of trainees under the British Medical Association (BMA) are working less than full time equivalent, while only 5.1% of Canadian trainees reported working less than 50 hours per week [36, 38]. Therefore, Canadian PGME and trainee unions may benefit from implementing policies developed by BMA for guidance on flexible work arrangements [38].

Furthermore, despite parents making up about 25% of trainees [39], only a single institution mentioned having emergency daycare onsite. Research suggests that increased flexibility may decrease work-to-family conflict [40], yet only two institutions had existing family-friendly policies, including the provision of family emergency days.

### Community engagement

Community engagement is a cornerstone of medical institutions’ commitment to social accountability [41]. Research has highlighted the profound influence of behavioural, social, and environmental factors on population health [42, 43]. Community engagement enables the creation of effective clinical and community programs and serves as an integral part of allyship by connecting with diverse communities and promoting inclusivity beyond institutional boundaries [44–47]. Despite these benefits, our analysis revealed that most PGME program websites lack publicly available EDI-promoting newsletters and communication regarding diversity-centered community engagement activities. However, internal newsletters with access restrictions requiring institutional login limited the extent to which community engagement efforts could be evaluated.

### Pathways to entry

While most PGME program websites explicitly mentioned transitional or re-entry programs, detailed qualification requirements and related application procedures were rarely addressed within available policies. Only two PGME websites highlighted their engagement with associations and professional organizations to promote diversity. To strengthen diversity initiatives, PGME may consider developing additional residency pathways, such as the Black applicant pathway to internal medicine at the University of Toronto [32]. These pathways can be implemented without changes to the existing application process administered through the Canadian Resident Matching System (CaRMS). For instance, in Toronto’s internal medicine Black and Indigenous student pathways, candidates self-identify their personal letter and are encouraged to reflect on how their identity influences their view of medicine and career goals [32, 33]. Additionally, establishing networking opportunities for visiting students from underrepresented groups may be beneficial [48].

### Limitations

Our study sample size was constrained by the number of Canadian Postgraduate Medical Education Offices, although it is representative of all medical schools in Canada. Data collection was restricted to publicly accessible websites, excluding alternative sources such as private forums, emails, or direct interaction with trainees or staff which prospective residents might utilize. Website assessments were conducted with a degree of subjectivity, employing a binary approach to evaluate the presence or absence of content. A comprehensive analysis and critique of available content fell beyond the scope of this study. This study focused on assessing PGME at an overarching level and did not delve into individual program activities, which may vary from the presented findings. It is important to note that more nuanced information may be available on individual residency program websites.

## Conclusion

The assessment of Canadian PGME websites reveals varying levels of commitment to EDI. Many PGME programs demonstrate a strong commitment to EDI through acknowledgement of EDI in mission statement, promotion of access to mental health services such as counselling, and deliberate harassment and discrimination policies. Areas of improvement include individualized and publicized EDI commitments from leaders, promotion of representation on interview panels, development of family friendly policies, deliberate recruitment of underrepresented groups, increased transparency of EDI initiatives through public engagement, and development of diversity recruitment pathways for medical residency training. Building a strong EDI presence online offers the opportunity to demonstrate commitment to EDI, and ultimately promotes the recruitment of a diverse post graduate trainee body.

## Supporting information

S1 Table Diversity audit tool EDI criteria selected for assessment of postgraduate medical education websites. (DOCX)

S2 Table EDI criteria assessed on postgraduate medical education websites. (DOCX)
