# Supplementary material for: Evaluating equity, diversity, and inclusion in Canadian Postgraduate Medical Education: A cross-sectional analysis of online content
Source: PLoS One. 2024 Aug 27;19(8):e0307584. doi: 10.1371/journal.pone.0307584 (PMC11349208; doi:10.1371/journal.pone.0307584)
Supplement: S1 Table — (DOCX) [file pone.0307584.s001.docx]

| **Diversity Audit Tool** | **Status** | **Reason for Exclusion** |  |
| --- | --- | --- | --- |
| **(1) Leadership and Governance** | | | |
| Does the board consider diversity in identifying and developing candidates? | Included |  |  |
| Do senior executive pro-actively communicate the importance of diversity inside and outside the organization? | Included |  |  |
| Do leaders reflect the composition of the workforce? | Excluded | Assessment Challenging - Composition difficult to quantify |  |
| Is there a diversity council? | Included |  |  |
| Is there a Chief Diversity Officer (CDO) at the Senior VP level with lines of authority? | Included |  |  |
| Has the business case for diversity been developed and widely communicated? | Included |  |  |
| Are explicit diversity goals and policies in place and communicated internally and externally? | Included |  |  |
| Are there well-developed mechanisms to handle employee complaints about harassment and discrimination? | Included |  |  |
| Is performance and compensation for managers tied to meeting diversity targets? | Excluded | Not Applicable - "Managers" similar positions difficult to quantify |  |
| Are there diverse leaders with profile internally and externally? | Excluded | Assessment Challenging - "Diversity" difficult to quantify |  |
| **(2) Strong and Transparent Human Resources Practices** | | | |
| **A. Recruitment** | | | |
| Are reviews of vacant positions undertaken to ensure that the qualifications required fit the demands of the job? | Excluded | Not Applicable - Positions not posted on PGME websites |  |
| Does the organization consider alternative pathways to positions? | Excluded | Repeat - Evaluated in Section 6 |  |
| Are vacant positions posted? | Excluded | Not Applicable - Positions not posted on PGME websites |  |
| Do recruiters specifically target underrepresented groups? | Included |  |  |
| Do all internship, co-op, and placement programs have diversity targets? | Excluded | Not Applicable - No co-op or internship roles |  |
| Are selection committee’s representative? | Included |  |  |
| Are bias-free interviewing processes used? | Included |  |  |
| **B. Performance Management** | | | |
| Is accountability for diversity targets and practices built into performance management systems? | Included |  |  |
| **C. Promotion** | | | |
| Does succession planning take into account diversity targets? | Excluded | Not Applicable |  |
| Are high potential employees from underrepresented groups given opportunities to take “stretch” assignments? | Excluded | Limited Information Disclosure - "Stretch" assignments informal |  |
| Are promotional opportunities and processes communicated openly and clearly to employees? | Excluded | Not Applicable - Positions not posted on PGME websites |  |
| Are career planning systems in place to support employees? | Excluded | Limited Information Disclosure - Formal process not well described |  |
| **D. Education, Training and Knowledge Building** | | | |
| Is diversity tracked in employee separations (retirements, dismissals, voluntary exits, layoffs)? | Excluded | Limited Information Disclosure |  |
| Are exit interviews conducted and are the results acted upon? | Excluded | Limited Information Disclosure |  |
| **E. Training and Developing Talent** | | | |
| Does orientation for new employees address diversity? | Excluded | Limited Information Disclosure - Not managed at PGME level |  |
| Do all employees receive mandatory training on diversity? | Excluded | Limited Information Disclosure - Not managed at PGME level |  |
| Do managers receive specialized training on diversity? | Excluded | Limited Information Disclosure - Not managed at PGME level |  |
| Do individuals involved in the hiring processes receive specialized training on hiring and bias-free hiring? | Excluded | Repeat – Section 2A |  |
| Are high-potential employees from diverse groups given access to specialized training and professional development that would enhance their ability to succeed? | Excluded | Not applicable – specialized training not managed at PGME level |  |
| Are provisions available for keeping employees current during/after parental leave? | Excluded | Repeat - Evaluated in Section 3 |  |
| Are customized training programs available for high potential employees? | Excluded | Not applicable – high potential training not managed at PGME level |  |
| Are formal mentoring/coaching programs (internal or external) provided? | Excluded | Not applicable – Specialty underrepresented groups mentorship not managed at PGME level |  |
| Are formal diversity networks supported? | Excluded | Repeat - Evaluated in Section 1 |  |
| **(3) Quality of Life and Organizational Culture** | | | |
| Are flexible working arrangements available? | Included |  |  |
| Are family-friendly policies in place including, for example, extended parental leave and family emergency days, elder care, support for parents traveling? | Included |  |  |
| Are on-site childcare and emergency day care services available? | Included |  |  |
| Are employee workloads and employer expectations managed? | Included |  |  |
| Do employees have access to coaching and counseling to help manage workload and stress? | Included |  |  |
| **(4) Measure and Track Diversity** | | | |
| Are there metrics on the participation of employees from underrepresented groups at each management level relative to the available labor force? | Excluded | Assessment Challenging - Composition difficult to quantify |  |
| Are there explicit diversity targets for participation and for employees from underrepresented groups in management? | Excluded | Not Applicable - "Management" similar positions difficult to quantify |  |
| Are there regular employee engagement surveys with self-reported demographic data? | Excluded | Not Applicable - Managed by the CaRMS process |  |
| Are equal pay audits conducted to ensure equal pay for work of equal value? | Excluded | Limited Information Disclosure - Payment models not managed at PGME level |  |
| Is performance benchmarked against others in the industry? | Excluded | Limited Information Disclosure - Diversity targets measured in Section 2B, formal benchmarking not typically performed |  |
| Are these results tracked and reported with feedback loops for action? | Excluded | Repeat - Covered in Section 2B |  |
| **(5) Integrate Diversity Across the Value Chain** | | | |
| Is diversity considered in designing and developing products? | Excluded | Not Applicable - No product |  |
| Does the organization consider and communicate the importance of diversity in its marketing and customer service programs? | Excluded | Not Applicable - No formal customer service and marketing department in PGME |  |
| Is the importance of diversity communicated in all its publications (internal and external)? | Included |  |  |
| Is the importance of diversity considered and communicated in media buys? | Excluded | Not Applicable - No formal Media Buys in PGME |  |
| Is the importance of diversity considered and communicated in philanthropic activities? | Included |  |  |
| Is the importance of diversity considered in government relations? | Excluded | Not Applicable - No formal government relations |  |
| Is the importance of diversity considered and communicated in procurement processes? | Excluded | Not Applicable - No formal procurement process |  |
| **(6) Develop the Pipeline** | | | |
| Is outreach to schools provided regarding opportunities and incentives in this industry? | Excluded | Not Applicable - Medical school and program-specific level |  |
| Does the organization participate with associations and professional organizations in programs to promote its commitment to diversity? | Included |  |  |
| Is the importance of diverse representation considered in partnerships with educational institutions (i.e. research, executive education, training and development)? | Excluded | Not Applicable - "Partnerships" with "educational institutions" not typical activities at PGME level |  |
| Does the organization collaborate and encourage development of re-entry and transitional programs? | Included |  |  |
| Does the organization work with institutions to support programs to encourage individuals from underrepresented groups to enter these kinds of jobs (i.e. hybrid programs and double majors)? | Excluded | Not Applicable - Program-specific |  |
| Does the organization ensure that all of its outreach activities in cooperation with educational institutions, government, and associations consider representation? | Excluded | Limited Information Disclosure - Limited details of outreach activities with indicated partners |  |
| Does the organization collaborate and support research and evaluation aimed at promoting effective diversity interventions? | Excluded | Limited Information Disclosure – Limited disclosure behind to develop pipeline |  |
| "Limited Information Disclosure" refers to insufficient information provided on websites to make an informed assessment.  "Not Applicable" indicates that certain criteria do not apply to PGME websites due to the nature of their operations or focus.  "Assessment Challenging" implies that while the criteria are relevant, assessing them accurately based on the available information is difficult.  "Repeat" denotes criteria that are evaluated in multiple sections of the Diversity Audit Tool. | | | |
