# Supplementary material for: Evaluating equity, diversity, and inclusion in Canadian Postgraduate Medical Education: A cross-sectional analysis of online content
Source: PLoS One. 2024 Aug 27;19(8):e0307584. doi: 10.1371/journal.pone.0307584 (PMC11349208; doi:10.1371/journal.pone.0307584)
Supplement: S2 Table — (DOCX) [file pone.0307584.s002.docx]

| **Criteria Assessed** | **Primary Content Evaluated*** |
| --- | --- |
| **Leadership and Governance** | Annual Report (2020 – 2021) |
|  | Organization Chart (PGME) |
|  | Committees Information Page |
|  | Strategic Plan |
|  | Discrimination and Harassment Policy |
|  | About Section (including message from the dean) |
| **Transparent Recruitment Practices** | Applicant Selection Policy |
|  | Interview Guidance Policy |
| **Program Accommodations and Culture** | Workplace Flexibility Policy |
|  | Pregnancy in Residency Policy |
|  | Wellness & Fatigue Policy |
|  | Accommodations Policy |
|  | Leave of Absence Policy |
|  | Mistreatment, Harassment, and Discrimination Policies |
| **Diversity in Community Engagement** | PGME Newsletters (June 2021 – July 2022) |
|  | Annual Report (2020 – 2021) |
| **Pathways to Entry** | Strategic Plan |
|  | Transfer Policy |
|  | Re-Entry Policy |
| *To be considered present, information had to be directly available on the PGME website, accessible through hyperlinks on the landing page, or available in publicly accessible linked documents. If links to documents or policies were nonfunctional, they were not considered present. Only directly present or hyperlinked policies and documentation on the PGME website were reviewed. Password-protected or institutionally restricted documents were excluded. In the absence of PGME-specific documents, relevant documents from the parent organization, Faculty of Medicine, were considered. If the 2020-2021 annual report was unavailable, the most recent accessible report was reviewed. | |
